# Validation of Sleep Measurements of an Actigraphy Watch: Instrument Validation Study

**DOI:** 10.2196/63529

**Published:** 2025-01-06

**Authors:** Mari Waki, Ryohei Nakada, Kayo Waki, Yuki Ban, Ryo Suzuki, Toshimasa Yamauchi, Masaomi Nangaku, Kazuhiko Ohe

**Affiliations:** 1 Department of Biomedical Informatics Graduate School of Medicine The University of Tokyo Tokyo Japan; 2 Department of Diabetes Metabolism and Endocrinology Tokyo Medical University Tokyo Japan; 3 Department of Diabetes and Metabolic Diseases Graduate School of Medicine The University of Tokyo Tokyo Japan; 4 Division of Nephrology and Endocrinology Graduate School of Medicine The University of Tokyo Tokyo Japan

**Keywords:** actigraphy, sleep, Motion Watch 8, iAide2, total sleep time

## Abstract

**Background:**

The iAide2 (Tokai) physical activity monitoring system includes diverse measurements and wireless features useful to researchers. The iAide2’s sleep measurement capabilities have not been compared to validated sleep measurement standards in any published work.

**Objective:**

We aimed to assess the iAide2’s sleep duration and total sleep time (TST) measurement performance and perform calibration if needed.

**Methods:**

We performed free-living sleep monitoring in 6 convenience-sampled participants without known sleep disorders recruited from within the Waki DTx Laboratory at the Graduate School of Medicine, University of Tokyo. To assess free-living sleep, we validated the iAide2 against a second actigraph that was previously validated against polysomnography, the MotionWatch 8 (MW8; CamNtech Ltd). The participants wore both devices on the nondominant arm, with the MW8 closest to the hand, all day except when bathing. The MW8 and iAide2 assessments both used the MW8 EVENT-marker button to record bedtime and risetime. For the MW8, MotionWare Software (version 1.4.20; CamNtech Ltd) provided TST, and we calculated sleep duration from the sleep onset and sleep offset provided by the software. We used a similar process with the iAide2, using iAide2 software (version 7.0). We analyzed 64 nights and evaluated the agreement between the iAide2 and the MW8 for sleep duration and TST based on intraclass correlation coefficients (ICCs).

**Results:**

The absolute ICCs (2-way mixed effects, absolute agreement, single measurement) for sleep duration (0.69, 95% CI –0.07 to 0.91) and TST (0.56, 95% CI –0.07 to 0.82) were moderate. The consistency ICC (2-way mixed effects, consistency, single measurement) was excellent for sleep duration (0.91, 95% CI 0.86-0.95) and moderate for TST (0.78, 95% CI 0.67-0.86). We determined a simple calibration approach. After calibration, the ICCs improved to 0.96 (95% CI 0.94-0.98) for sleep duration and 0.82 (95% CI 0.71-0.88) for TST. The results were not sensitive to the specific participants included, with an ICC range of 0.96-0.97 for sleep duration and 0.79-0.87 for TST when applying our calibration equation to data removing one participant at a time and 0.96-0.97 for sleep duration and 0.79-0.86 for TST when recalibrating while removing one participant at a time.

**Conclusions:**

The measurement errors of the uncalibrated iAide2 for both sleep duration and TST seem too large for them to be useful as absolute measurements, though they could be useful as relative measurements. The measurement errors after calibration are low, and the calibration approach is general and robust, validating the use of iAide2’s sleep measurement functions alongside its other features in physical activity research.

## Introduction

### Background

The iAide2 (Tokai) is a physical activity monitoring system that measures intensity levels, step counts, temperature, and pulse rate [1,2] to assess activity and sleep, with minute-by-minute assessment of wake/sleep [3,4]. It is based on TDK’s Silmee W20/W22 actigraphs [4-6] used in previous research [5,7-9]. The iAide2 supports long trials (via rechargeability and off-watch data storage) and timely behavior feedback (via a wireless EVENT button and near–real-time access to data). Its sleep measurements are not well validated; Kimura et al [7] compared its predecessor, the W20, to video-monitoring sleep assessment (which is itself unvalidated), and reported 5 sleep duration values, with detailed methodology not given [7].

Actigraphs are commonly validated against polysomnography (PSG) or another actigraph. PSG, though imperfect, is the gold standard of sleep measurement [10]. PSG’s complexity requires sleep in a laboratory [11], which is costly and unrepresentative of free-living sleep. Watch-based actigraphy can efficiently collect multiple nights’ data in a natural environment [12,13].

### Objective

We assessed iAide2-measured sleep duration and total sleep time (TST) in participants without sleep disorders and added calibration when needed.

## Methods

### Approach

To assess free-living sleep, we validated iAide2 against a second actigraph, the MotionWatch 8 (MW8; CamNtech Ltd) [14], using methods similar to other actigraph-to-actigraph comparisons [11,15-17]. The MW8 is validated against PSG [18-20], with high correlations (*r*=0.920) and no significant bias (7.0 minutes) for TST [19]. Researchers have used the MW8 to assess sleep in people without sleep disorders [21-23]. We used validated MW8 settings (mode 1, 30-second epochs, and a threshold of 20 [24,25]).

### Data Collection

We collected data from a convenience sample of members of the Waki DTx Laboratory at the Graduate School of Medicine, University of Tokyo. The laboratory is investigating lifestyle interventions to improve sleep and exercise and identified the iAide2 as a candidate measurement device. This validation study assessed the accuracy of iAide2 sleep monitoring in 6 individuals without known sleep disorders. They wore both devices on the nondominant arm, with the MW8 closest to the hand, all day except when bathing.

The participants used the MW8 EVENT-marker button to record bedtime (when they began trying to sleep) and risetime (when they stopped trying to sleep). The duration between these times is time in bed (TIB). Our MW8 and iAide2 assessments both used these nonactigraphy measurements.

MotionWare (version 1.4.20; CamNtech Ltd) provided sleep onset (“Fell Asleep” in MotionWare, the earliest time after bedtime when the participant was asleep), sleep offset (“Woke Up,” the latest time prior to risetime when the participant transitioned from asleep to awake), and TST (the sum of all minutes of sleep between sleep onset and offset) [25]. From these measurements, we derived sleep onset latency (the duration between bedtime and sleep onset), sleep offset latency (the duration between sleep offset and risetime), and sleep duration (the duration between sleep onset and sleep offset).

We used a similar process with the iAide2, using iAide2 software (version 7.0). We defined sleep onset as the time of the first sleep after bedtime. Similarly, we defined sleep offset as 1 minute after the time of final sleep prior to risetime. We defined TST as the sum of minutes asleep within the sleep duration period.

### Statistical Analysis

We compared measurements using intraclass correlation coefficients (ICCs; 2-way mixed effects, absolute agreement, single measurement, unless otherwise specified) [26,27]. We used the *irr* package 0.84.1 in R for point estimation of ICC and calculating 95% CIs, and R (version 4.2.1) for calibration.

### Ethical Considerations

The University of Tokyo School of Medicine institutional review board (2024137NI) approved this study, which abided by the Declaration of Helsinki. Participants provided verbal consent for data collection upon recruitment. All participants provided written consent for the use of their data. Participants’ data were anonymized. No compensation was provided.

## Results

### Participants

**Figure 1:**
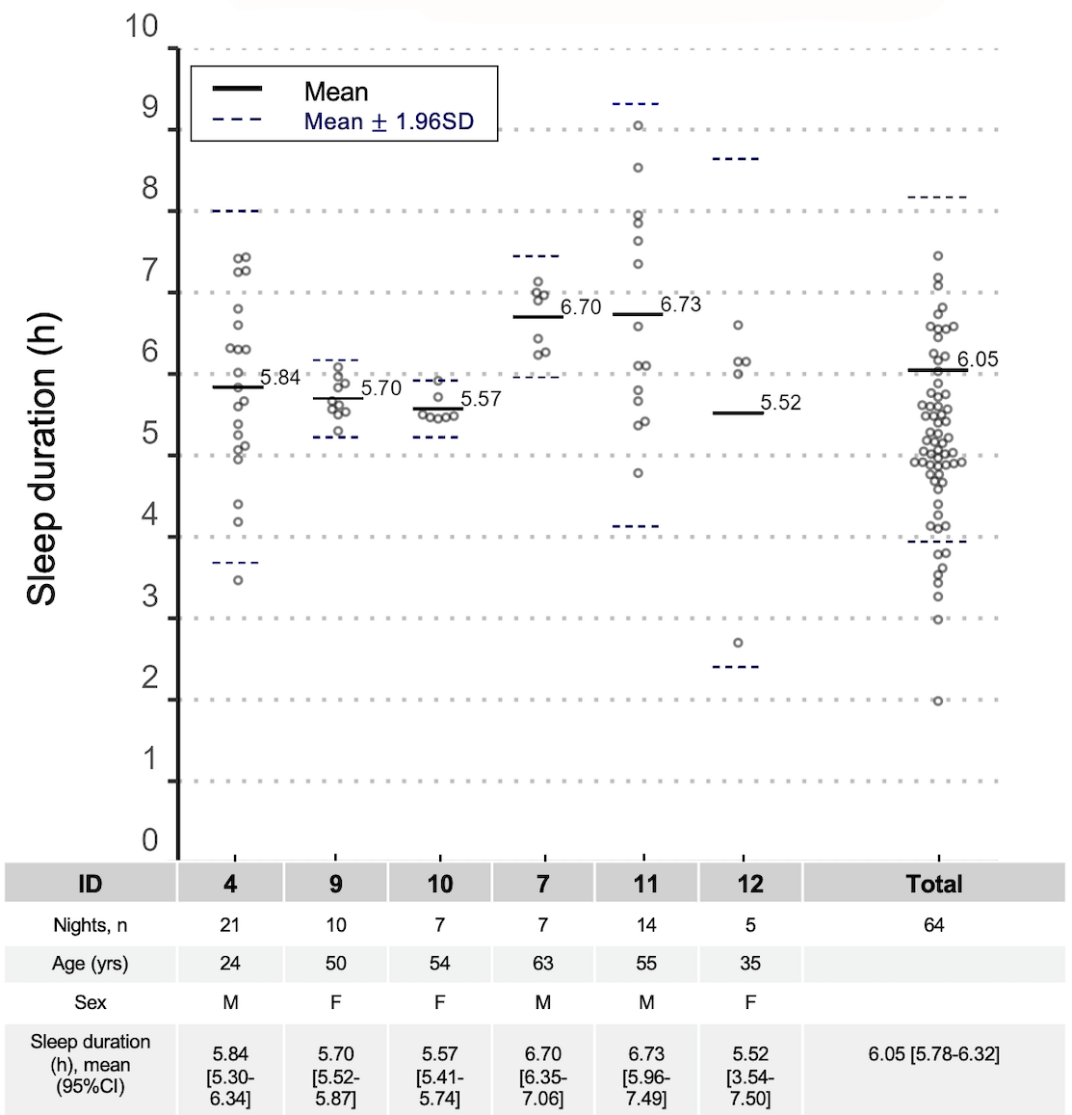
Participants’ information and sleep duration measured using the MotionWatch 8 actigraph (MW8; 6 participants, 64 nights).

We recruited 6 participants (aged 24-63 years) and recorded data for 64 nights (Figure 1). The number of nights per participant varied from 5 to 21. The average nightly sleep duration via MW8 per participant varied from 5.52 to 6.73 hours, and the participants differed in the regularity of their sleep durations.

### Measurements

**Figure 2:**
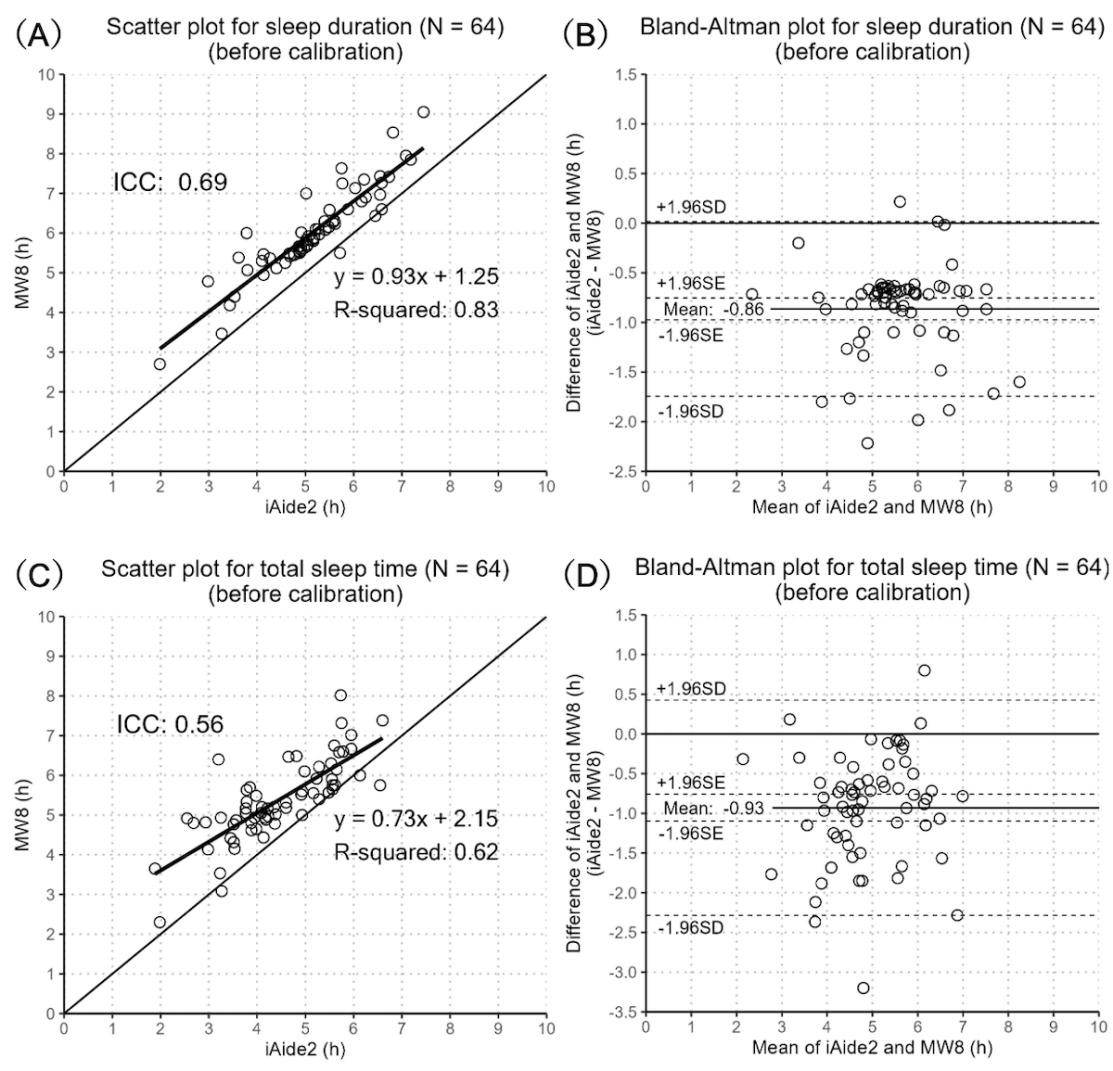
Scatter plot and Bland-Altman plot for measured sleep duration and total sleep time using iAide2 and the MotionWatch 8 actigraph (MW8; 64 nights).

The iAide2 underestimated duration relative to the MW8 and showed moderate ICC of 0.69 (95% CI –0.07 to 0.91; Figure 2A) [26]. Relative performance was excellent (ICC=0.91, 95% CI 0.86-0.95; 2-way mixed effects, consistency, single measurement) [26]. The mean, maximum, and minimum differences between iAide2 and MW8 were –0.86, 0.22, and –2.22 hours, respectively (Figure 2B).

Similarly, the iAide2 underestimated TST and showed moderate ICC of 0.56 (95% CI –0.07 to 0.82; Figure 2C) and a good consistency ICC (2-way mixed effects, consistency, single measurement) of 0.78 (95% CI 0.67-0.86). The mean, maximum, and minimum differences between iAide2 and MW8 were –0.93, 0.80, and –3.20 hours, respectively (Figure 2D).

### Calibration

Our iAide2 data showed very small sleep offset latencies (mean 0.03 hours) but significant overestimates of sleep onset latency, so we corrected the sleep duration by the difference between the iAide2-measured sleep onset latency and an iAide2 sleep onset latency calibrated to the MW8 measurements via ordinary least squares (OLS). We added a small (<5 minute) bias correction, selected to optimize ICC, to account for small offset latency biases. To prevent physically impossible results, we limited the calibrated duration to be no longer than the TIB. The resulting calibration equation is D’ = min {0.59S + D + 0.26, TIB} (hours), where D is the measured duration, S is the measured sleep onset latency, and D’ is the calibrated duration.

For TST, we used OLS to calibrate the measured TST value, limiting the result to be not greater than the calibrated sleep duration. The resulting equation is T’ = min {0.73T + 2.15, D’} (hours), where T is the measured TST and T’ is the calibrated TST.

**Figure 3:**
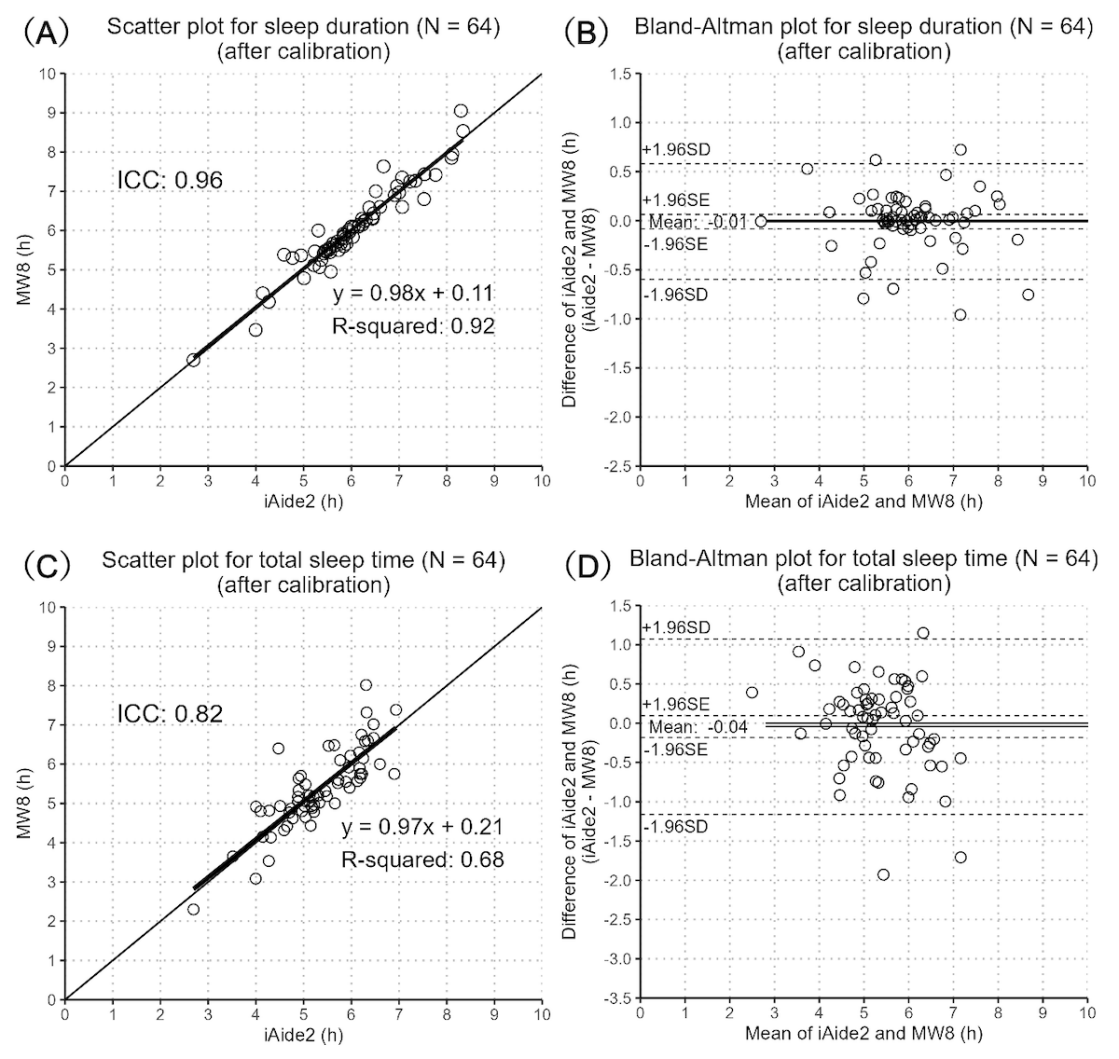
Scatter plot and Bland-Altman plot for calibrated sleep duration and total sleep time using iAide2 and the MotionWatch 8 actigraph (MW8; 64 nights).

Calibration greatly improved accuracy. Sleep duration had excellent ICC (0.96, 95% CI 0.94-0.98; Figure 3A). The mean difference almost disappeared (–0.01 hours) and the difference between the maximum (0.72 hours) and minimum (–0.96 hours) values decreased (Figure 3B). TST similarly had greatly improved accuracy, with good ICC (0.82, 95% CI 0.71-0.88; Figure 3C). The mean difference became almost zero (–0.04 hours) and the difference between the maximum (1.15 hours) and minimum (–1.93 hours) values decreased (Figure 3D).

Because our sample size is small with a varying number of nights per participant, we conducted a sensitivity analysis to determine how the ICC agreement rate changes based on the included participants. First, to show that our ICCs are not sensitive to the exact individuals, we performed trials excluding the data of one participant at a time, recalibrating for each trial. The calibrated ICC range was 0.96-0.97 for sleep duration, effectively unchanged from 0.96 in the baseline analysis, and 0.79-0.86 for TST, similar to 0.82 in the baseline analysis, demonstrating that our results are not highly influenced by the variability in number of nights per participant. To demonstrate the generality of our calibration equations, we applied the calibration derived from all participants’ data to trials excluding one participant at a time. The ICC range was 0.96-0.97 for sleep duration and 0.79-0.87 for TST, again similar to baseline analysis values, showing that our calibration has high generality and is not highly dependent on any one participant’s data.

## Discussion

### Principal Findings

The absolute accuracies of uncalibrated iAide2 measurements for sleep duration (ICC=0.69) and TST (ICC=0.56) are relatively poor. Uncalibrated data could be useful in applications needing relative measurements, with good values for the consistency version of the ICC (2-way mixed effects, consistency, single measurement) of 0.91 for sleep duration and 0.78 for TST.

The calibrated measurements have excellent absolute accuracy. The ICCs for sleep duration (0.96) and TST (0.82) are comparable to or better than those reported previously (0.67 and 0.68 for sleep duration and 0.77 and 0.84 for TST [28,29]). Sensitivity analysis revealed essentially unchanged performance when applying this calibration to data while removing one participant at a time and when recalibrating while removing one participant at a time. The calibration methods are simple and have good statistical bases. The calibrated iAide2 measurements of sleep duration and TST seem usable in a wide range of applications.

### Limitations

The MW8, our validation standard, has its own errors, with a reported underestimation of sleep onset latency (mean difference 8.9 minutes) and overestimation of TST (mean difference 47.1 minutes) relative to PSG [30]. Validation against other standards, including PSG, is warranted.

We used more nights (N=64) than some PSG-based validation studies (N=20, 37, and 38) but fewer than actigraph-based research (N=140) [17,18,31,32]. Our convenience sample was small (N=6); however, it included a mix of ages, sex, and sleeping regularity. Our results may not fully apply to other populations; and larger studies are warranted.

### Conclusions

The iAide2 has interesting measurement capabilities with convenient features, and validating its sleep measurement accuracy adds to its usefulness. The absolute accuracies are low, but good relative accuracy may be suitable for some applications. Its postcalibration accuracies are excellent and well validated against the MW8.

## Abbreviations

ICC: intraclass correlation coefficient
MW8: MotionWatch 8 actigraph
OLS: ordinary least squares
PSG: polysomnography
TIB: time in bed
TST: total sleep time

## Data Availability

The data that support the findings of this study are available from the corresponding author upon reasonable request for noncommercial purposes.
